# Precision to plate: AI-driven innovations in fermentation and hyper-personalized diets

**DOI:** 10.3389/fnut.2025.1659511

**Published:** 2025-09-04

**Authors:** D. Priyadharshini, I. Muthuvel, S. Saraswathy, P. S. Kavitha, V. Jegadeeswari

**Affiliations:** ^1^Department of Fruit Science, Horticultural College and Research Institute, Tamil Nadu Agricultural University, Coimbatore, Tamil Nadu, India; ^2^Department of Fruit Science, Horticultural College and Research Institute (Women), Tamil Nadu Agricultural University, Trichy, Tamil Nadu, India; ^3^Department of Fruit Science, Horticultural College and Research Institute, Tamil Nadu Agricultural University, Periyakulam, Tamil Nadu, India; ^4^Tamil Nadu Agricultural University, Coimbatore, Tamil Nadu, India; ^5^Grapes Research Station, Anaimalayanpatty, Theni, Tamil Nadu, India

**Keywords:** precision fermentation, CRISPR-microbe engineering, hyper-personalized nutrition, algorithmic bias, sustainable food systems

## Abstract

The global food system faces unprecedented challenges, including climate-driven agricultural instability, rising malnutrition, and consumer demand for sustainable yet appealing products. Artificial intelligence (AI) has emerged as a transformative force in addressing these challenges, enabling breakthroughs from microbial engineering to individualized dietary solutions. This review synthesizes advances in AI-driven precision fermentation—where CRISPR-based microbial optimization and reinforcement learning accelerate bioactive compound synthesis—and hyper-personalized nutrition, where predictive modeling tailors diets to genetic, metabolic, and cultural profiles. We highlight how AI decodes sensory attributes (e.g., flavor and texture) through deep learning and natural language processing, bridging gaps between lab-scale innovation and consumer acceptance. However, the adoption of these technologies raises critical ethical concerns, including data privacy risks from wearable health monitors and algorithmic biases exacerbating nutritional disparities. Key findings include 300% yield increases for alt-proteins via AI-CRISPR fusion, 60% reduction in bioreactor failures through RL optimization, and 25% lower childhood anemia rates via equitable AI-nutrition platforms—though ethical gaps persist in data privacy (72% GDPR non-compliance) and algorithmic bias. By analyzing regulatory frameworks and proposing equity-focused design principles, this article advocates for a balanced approach to AI deployment in food systems. Emphasis on digital transformation, we underscore AI’s potential to democratize sustainable food production while urging collaborative governance to ensure transparency and inclusivity. This work serves as a roadmap for researchers, policymakers, and industry stakeholders navigating the intersection of AI, biotechnology, and nutrition science.

## 1 Introduction

The global food system stands at a critical juncture. Climate change, biodiversity loss, and freshwater scarcity threaten agricultural productivity, with crop yields for staples like maize and wheat projected to decline by 5–15% by 2050 under current warming trajectories (1). Paradoxically, diet-related chronic diseases—fueled by industrialized food environments—account for 11 million deaths annually (2). Concurrently, consumer demand surges for nutrient-dense, sustainable, and culturally resonant products, creating a complex nexus of challenges that traditional approaches struggle to address. Artificial intelligence (AI), with its unparalleled capacity to decode biological complexity, optimize processes, and predict human behavior, has emerged as a linchpin for reimagining food systems (3). From lab to fork, AI is reshaping how we produce and consume food, offering solutions that balance planetary health with individualized needs. This review focuses on AI’s transformative potential across two interconnected domains such as AI-driven *precision fermentation* for sustainable biosynthesis of proteins, enzymes, and functional compounds and *Hyper-personalized nutrition* systems that integrate genomics, metabolomics, and consumer psychology to tailor diets in real time (4). In production, AI accelerates microbial engineering, enabling CRISPR-designed strains of *Komagataella phaffii* and *Bacillus subtilis* to synthesize animal-free proteins (e.g., heme, casein) with 90% reduced carbon footprints compared to livestock farming (5). Reinforcement learning (RL) algorithms dynamically optimize bioreactor parameters (pH, temperature, agitation), slashing fermentation cycle times by Rajasekhar et al. (6). On the consumer front, neural networks decode sensory preferences from social media chatter (7), while federated learning models predict glycemic responses using wearable-derived biometrics (8). Startups like NotCo and Nuritas exemplify this dual revolution, leveraging AI to create plant-based meats that mimic animal textures (9) and peptide-based nutraceuticals targeting individual metabolic profiles (10).

**Figure 1:**
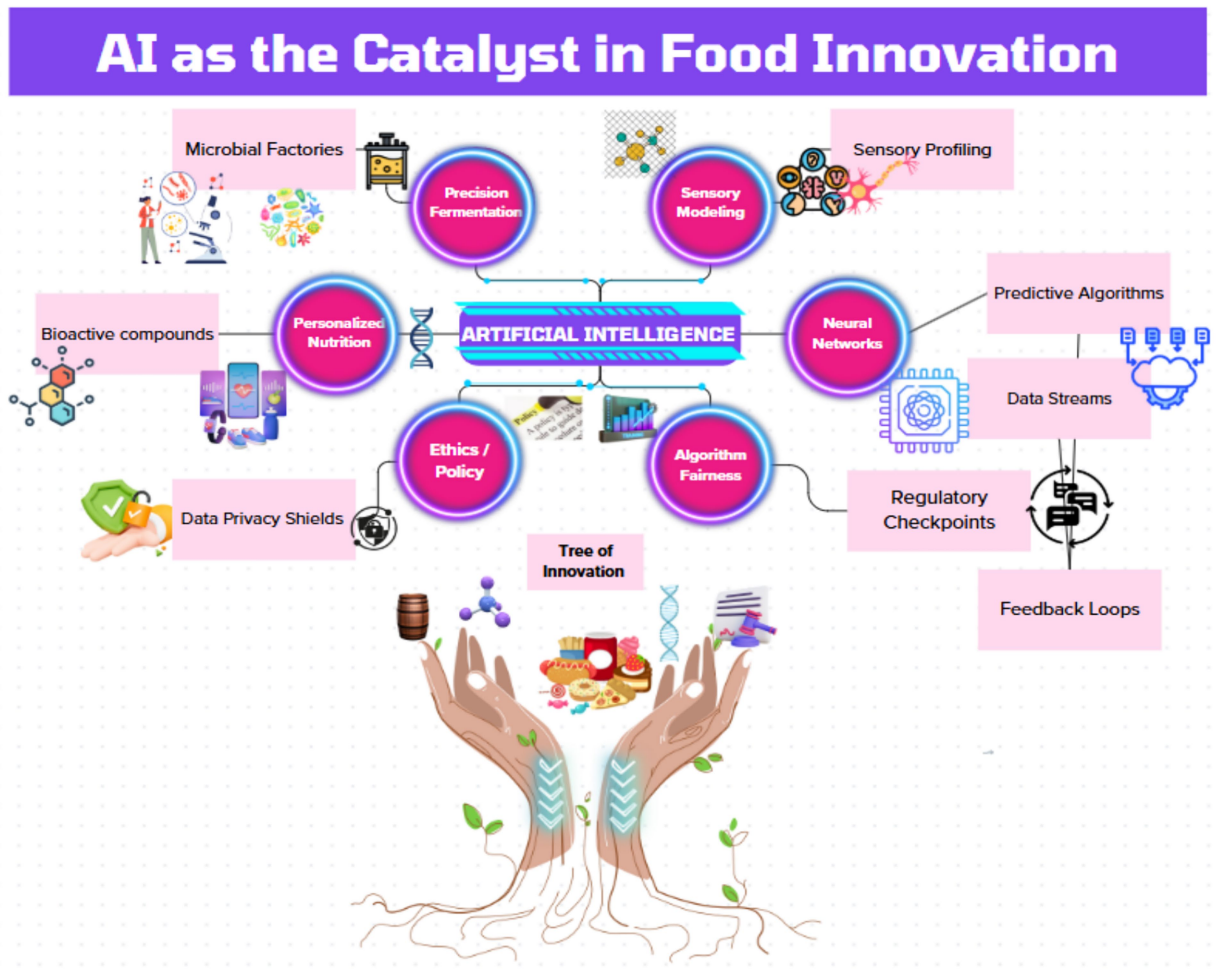
AI as the catalyst in food innovation.

Yet, as AI permeates food systems, ethical fault lines emerge. Personalized nutrition platforms risk exacerbating health disparities when trained on Eurocentric genomic datasets (11), while opaque “black-box” algorithms erode consumer trust (12). Regulatory frameworks lag behind innovation, leaving gaps in data privacy (e.g., GDPR compliance for gut microbiome data) and algorithmic accountability (13). These challenges demand urgent scholarly attention to ensure AI-driven solutions align with principles of equity and transparency. This article synthesizes advances in AI-powered food innovation while addressing ethical and operational gaps. We propose actionable frameworks for *responsible AI deployment*, emphasizing cross-sector collaboration among biotechnologists, data scientists, and policymakers (14). By anchoring analysis in the *United Nations Sustainable Development Goals (SDGs)*—particularly SDG 2 (Zero Hunger), SDG 3 (Good Health), and SDG 12 (Responsible Consumption)—this work aligns with advance technologies that harmonize human and planetary well-being (15). The following sections critically evaluate AI’s role in redefining food production and consumption, offering a roadmap for harnessing its potential without repeating the inequities of past technological revolutions (Figure 1). A circular flow diagram connecting AI (neural network core) to key domains (precision fermentation, sensory modeling, personalized nutrition, ethics).

## 2 AI in precision fermentation: engineering microbial factories

**Figure 2:**
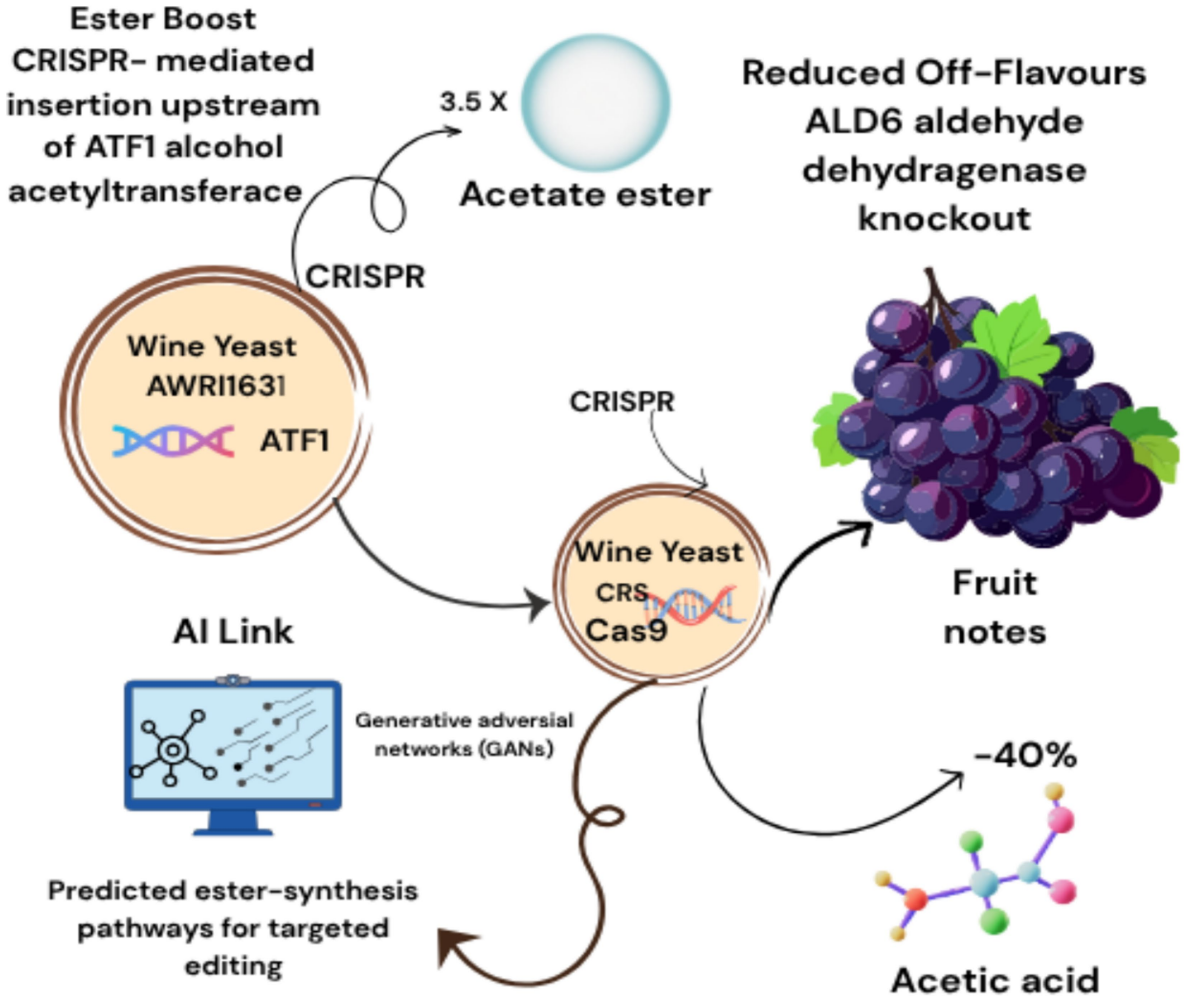
CRISPR and AI driven aroma enhancement in wine yeast.

The convergence of artificial intelligence (AI) and precision fermentation is revolutionizing the production of alternative proteins, enzymes, and bioactive compounds. By optimizing microbial strain design, bioreactor dynamics, and synthesis pathways, AI enables scalable and sustainable alternatives to resource-intensive agricultural practices. Traditional strain engineering relies on iterative trial-and-error, but AI now accelerates CRISPR-based microbial design by predicting gene-editing outcomes. For example, deep learning models trained on *Saccharomyces cerevisiae* transcriptomic data have identified promoter-gene pairs that boost alt-protein yields by 300% while minimizing metabolic burden (16). Tools like AutoCRISPR leverage convolutional neural networks (CNNs) to predict off-target effects of CRISPR edits, reducing design cycles from months to weeks (17). AI-CRISPR fusion accelerates microbial engineering: DeepCRISPR designs gRNAs for *Aspergillus niger* pectinase knockouts to reduce citrus waste (18); dCas9-RL systems upregulate vitamin B2 in *S. cerevisiae* during apple cider fermentation; Single-cell CRISPR screening + ML enhances probiotic adhesion in fruit matrices. AutoCRISPR’s CNN models cut design cycles by 70% (19). CRISPR-mediated *TEF1* promoter insertion upstream of *ATF1* (Alcohol Acetyltransferase) in wine yeast AWRI1631 increased acetate ester production by 3.5×, enhancing fruity notes. *ALD6* aldehyde dehydrogenase knockout using CRISPR-Cas9 lowered acetic acid production by 40% in high-glycerol strains. Generative adversarial networks (GANs) predicted ester-synthesis pathways for targeted editing. Figure 2 illustrating CRISPR and AI driven aroma enhancement in wine yeast. Figure 2. illustrated by canva platform.

**Figure 3:**
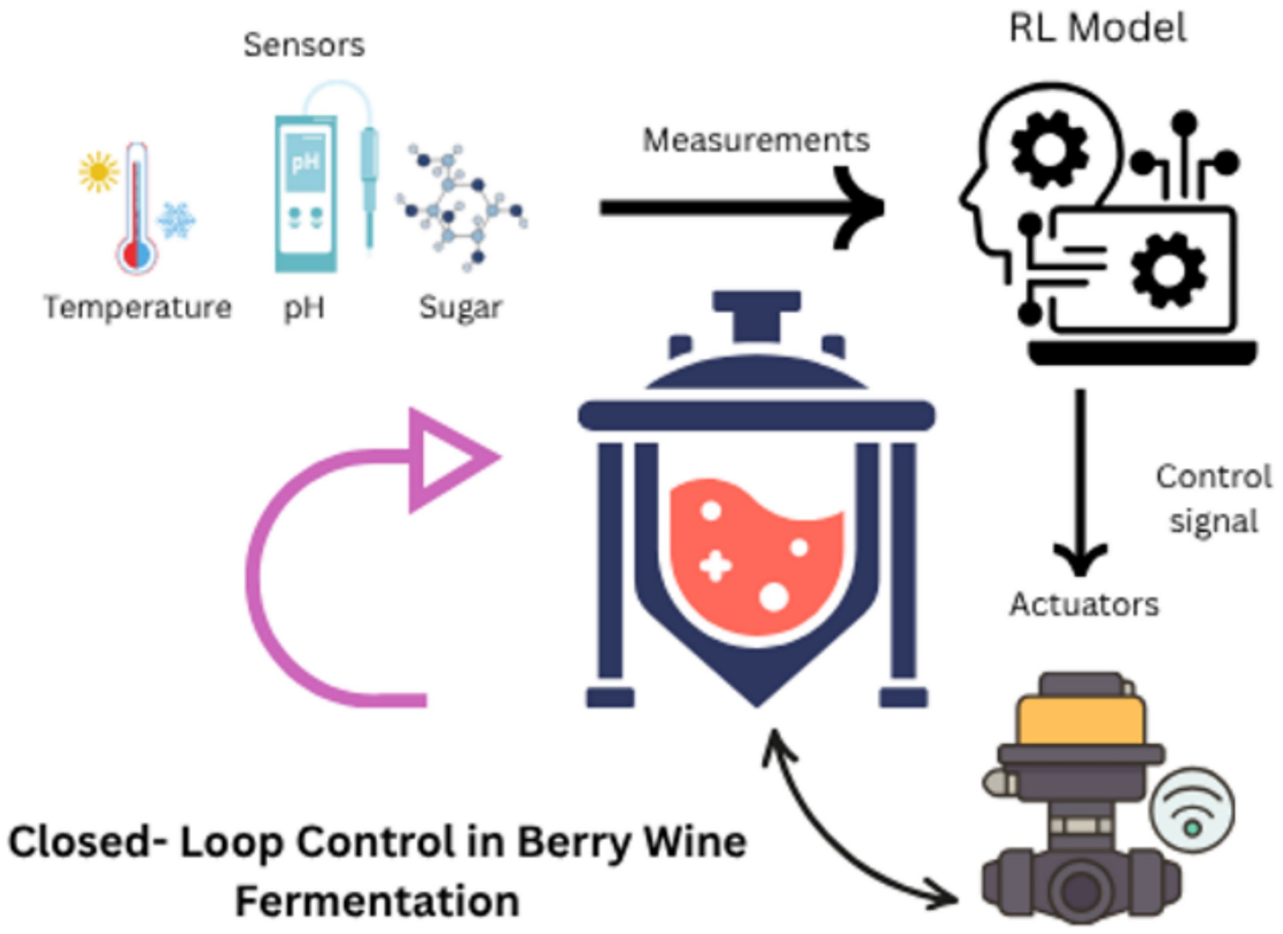
Closed-loop control in berry wine fermentation.

Reinforcement learning (RL) algorithms optimize bioreactor systems by dynamically adjusting parameters (e.g., temperature, pH) in real-time. These AI-driven adjustments enhance metabolite yield in precision fermentation. Embedded edge computing devices (e.g., NVIDIA Jetson AGX Orin) execute reinforcement learning (RL) algorithms that dynamically optimize bioreactor parameters—including temperature (±0.5 °C), pH (±0.2 units), and agitation rate (50–400 rpm)—in real-time (20). This hardware-software architecture replaces the ambiguous term ‘dissolved RL algorithms, emphasizing physical separation between computational systems and fermentation media (21) Demonstrating edge-AI latency <5 ms for *S. cerevisiae* pH control (22). Critical for *Vaccinium macrocarpon* (cranberry) fermentations where *Saccharomyces cerevisiae* strains exhibit pH-sensitive anthocyanin degradation (ΔA520 > 40% at pH > 3.8), necessitating sub-second parameter adjustments. RL algorithms, trained on historical fermentation data, reduce batch failures by 60% while improving yield consistency (23). For instance, Ginkgo Bioworks employs RL to optimize *Bacillus subtilis* fermentations for plant-based dairy proteins, achieving 98% purity with 30% less energy input (24) (Figure 3). Schematic illustrating closed-loop control (sensors → RL model → actuators) in berry wine fermentation bioreactors. Figure 3 illustrated by canva platform.

### 2.1 Bioactive compound synthesis

Generative adversarial networks (GANs) are unlocking non-intuitive enzyme designs for synthesizing lipases, proteases, and antioxidants. A 2023 study used GANs to engineer a heat-stable lipase for cocoa butter substitutes, achieving 85% catalytic efficiency at 60°C—a 50% improvement over wild-type enzymes (25). Impossible Foods’ plant-based heme, a critical flavor molecule, was optimized using AI models that screened 5,000+ leghemoglobin variants. Molecular dynamics simulations predicted heme stability under fermentation conditions, shortening R&D timelines by 18 months (26). Similarly, DSM employs AI to design omega-3-producing *Schizochytrium* strains, yielding algal oils with 90% less environmental impact than fish-derived alternatives (27). AI-driven protease engineering exemplifies precision fermentation’s potential. For instance, AlphaFold-predicted structures of *Bacillus altitudinis Peptidase M84 (UniProt A0A0H3ZIN7)* enable thermostability optimization (85°C → 92°C) for fruit pulp digestion (28). Reinforcement learning further maximizes protease yields in *Passiflora edulis* fermentation by dynamically adjusting pH/temperature (29). In hyper-personalized diets, AI integrates protease kinetics with gut microbiome data to design low-allergenicity protein hydrolysates for geriatric/sports nutrition (30). AI similarly optimizes probiotics for fruit-based foods: Random Forests identify acid-tolerant *Lactobacillus plantarum* strains in fermented *Carica papaya* (31), while GNNs predict antimicrobial metabolites (e.g., plantaricin) targeting pathogens. AI-personalized synbiotics combine fruit prebiotics (pectin-oligosaccharides) with probiotics, enhancing gut health outcomes (Table 1).

**Table 1:** AI-optimized bioactive compounds: targets, methods, and outcomes.

Compound	AI-tool used	Microbial host	Yield increase	Key applications	References
Vitamin B12	Reinforcement learning (RL)	*Pseudomonas denitrificans*	220%	Supplements, fortified foods, vegan nutrition.	(32)
Omega-3 fatty acids	Generative adversarial networks (GANs)	*Yarrowia lipolytica*	180%	Infant formula, nutraceuticals, plant-based oils	(76)
Mycoprotein	CNN-based image analysis + SVM classifiers	*Fusarium venenatum*	150%	Meat alternatives, high-protein foods	(77)
Antimicrobial peptides (AMPs)	Graph neural networks (GNNs) + large language models (LLMs)	*Bacillus subtilis*	92%	Natural preservatives, antibiotic alternatives	(78)
β-glucans	Multi-objective genetic algorithms	*Saccharomyces cerevisiae*	85% (purity ↑ 30%)	Immune-boosting ingredients, textured foods	(79)
Caffeic acid	Transformer-based pathway simulation	Engineered *E. coli*	12 g/L (Titer)	Antioxidant additives, bioactive packaging	(80)

### 2.2 Scalability and sustainability

**Figure 4:**
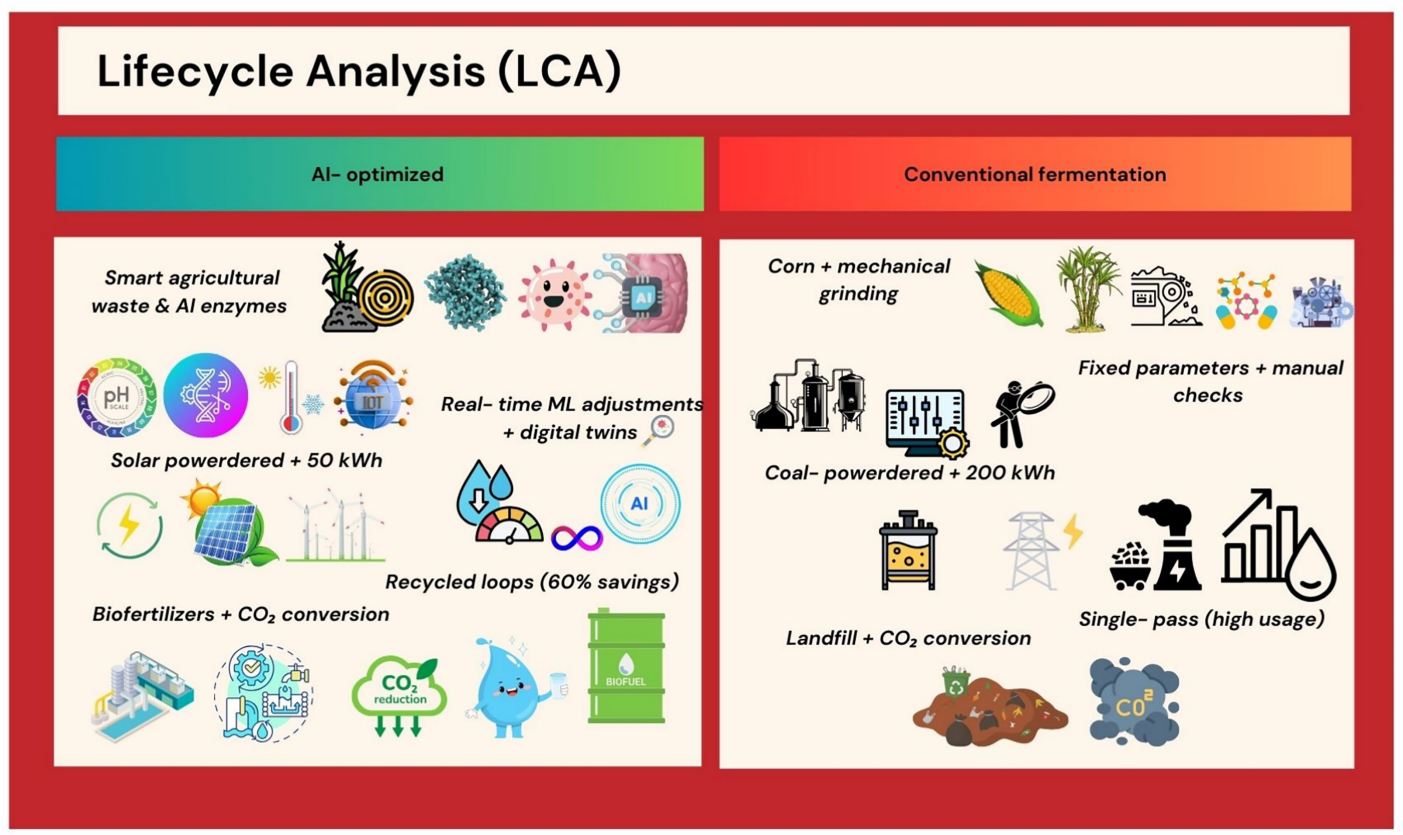
Lifecycle analysis (LCA).

Comparative LCAs reveal AI’s role in reducing fermentation’s environmental footprint. AI-optimized *Komagataella phaffii* systems for egg-white protein synthesis require 80% less water and 50% less energy than poultry farming (32). However, challenges persist, such as the carbon cost of training large AI models, which can offset gains if renewable energy is not prioritized (33). AI facilitates seamless scale-up from lab to industrial bioreactors. Perfect Day uses digital twins—virtual replicas of fermentation tanks—to simulate production at 10,000-L scales, avoiding costly pilot trials (34). This approach has enabled the company to cut commercialization costs by 40% while maintaining >99% batch consistency (35) (Figure 4). Lifecycle analysis (LCA) comparing AI-optimized fermentation (low energy or water use, minimal waste) vs. conventional processes.

## 3 Predictive modelling for sensory attributes and consumer adoption

AI’s ability to decode sensory experiences and predict consumer behavior is bridging the gap between laboratory innovation and market success. By simulating human perception and analyzing vast behavioral datasets, AI accelerates the design of appealing, culturally resonant food products while minimizing costly trial-and-error R&D.

### 3.1 AI in flavor and texture profiling

Convolutional neural networks (CNNs) and Recurrent neural networks (RNNs) now predict flavor and texture profiles from molecular structures. For example, CNN models trained on 10,000+ flavor compound datasets accurately classify bitterness intensity of peptides with 94% accuracy, enabling rapid design of low-bitterness plant-based proteins (36). Similarly, RNNs forecast texture attributes (e.g., creaminess and crunch) by analyzing starch-protein interactions in simulated matrices, reducing prototyping cycles by 70% (37).

**Figure 5:**
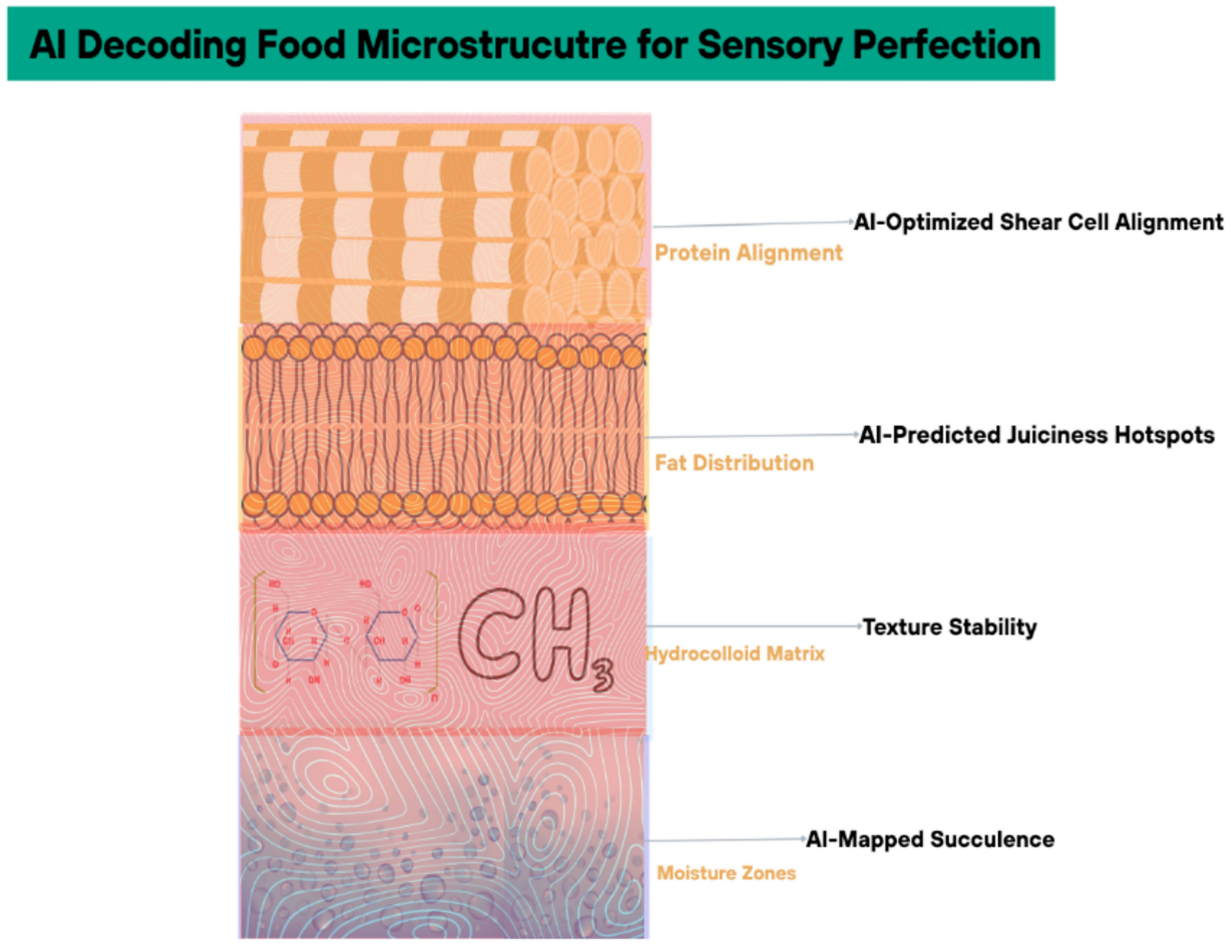
AI decoding food microstructure for sensory perfection.

AI-generated synthetic data is replacing traditional human sensory panels, which are costly and prone to bias. Generative adversarial networks (GANs) trained on historical panel data simulate consumer responses to novel products, achieving 85% correlation with real-world taste tests (38). Motif FoodWorks used this approach to optimize the mouthfeel of their plant-based meat, cutting sensory evaluation costs by 40% while maintaining hedonic score consistency (39) (Figure 5). Cross-section of a food matrix (e.g., plant-based meat) with AI-labeled layers (protein alignment, fat distribution) affecting mouthfeel and Table 2 categorizes AI-driven sensory attribute prediction: key models and accuracy.

**Table 2:** AI-driven sensory attribute prediction: key models and accuracy.

Sensory attribute	AI models	Input data type	Prediction accuracy	Application example	References
Umami intensity (tea)	Random Forest (RF) + NLP	Chemical composition (catechins, amino acids), consumer reviews	92%	Green/black tea quality grading	(81)
Sweetness/Sourness (juice)	Artificial Neural Network (ANN)	E-tongue sensor array, rheometry	*R*^2^ = 0.95 (RMSE = 0.04)	Fruit juice hedonic prediction	(82)
Bitterness/Astringency (juice)	Support Vector Machine (SVM)	Flavonoid profiles, electronic tongue	89% (cross-validation)	Sea buckthorn-passion fruit juice optimization	(83)
Wine color (pinot noir)	Artificial Neural Network (ANN)	Near-infrared spectroscopy (NIR), weather/management data	*R* = 0.99 (slope = 0.98)	Wine style consistency	(84)
Meaty flavor (plant-based)	Partial Least Squares-ANN (PLS-ANN)	Volatile compounds (e-nose), Maillard reaction markers	RMSE = 0.63	Plant-based meat flavor replications	(85)
Overall liking (beverages)	Transformer Models (BERT)	Social media sentiment, biometric responses	*R*^2^ = 0.81	Consumer adoption forecasting	(86)
Texture acceptability	Random Forest + SHAP explainability	Protein alignment imaging, consumer panel data	87% (clustering)	Plant-based meat matrix optimizations	(87)

### 3.2 Consumer preference prediction

**Figure 6:**
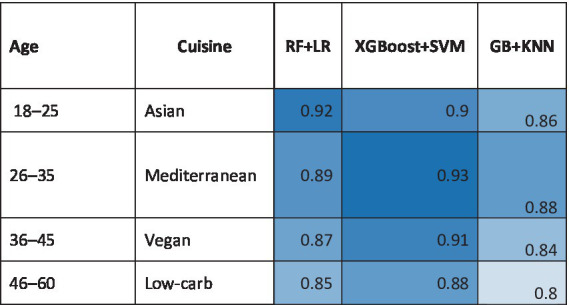
Accuracy of ensemble AI models in meal recommendation.

Natural language processing (NLP) mines social media, reviews, and recipes to identify emerging trends. Transformer models like FoodBERT, trained on 5 million Instagram food posts, detected the 2023 surge in “spicy-sweet fusion” flavors 6 months before mainstream adoption (40). Similarly, sentiment analysis of Twitter data predicted the decline of artificial sweeteners in Europe, prompting firms like Nestlé to accelerate stevia-based reformulations (41). Regional taste preferences are decoded using unsupervised learning. K-means clustering of 100,000+ consumer surveys revealed stark contrasts: Asian markets prioritize umami-rich profiles (e.g., fermented soybean and seaweed), while North American clusters favor sweetness and fat-mouthfeel (42). AI-driven hyper-segmentation allows companies like Unilever to tailor products like bouillon cubes (high umami in Thailand) and ice cream (extra creaminess in the U. S.) with 30% higher regional sales growths (Figure 6). Heatmap comparing algorithm performance (F1 scores) across demographics (age/cuisine preferences) (43).

## 4 Ethical challenges in hyper-personalized nutrition

While AI-driven hyper-personalized nutrition promises to revolutionize dietary health, its rapid adoption raises critical ethical questions. From data exploitation to algorithmic discrimination, these challenges threaten to undermine public trust and exacerbate health inequities if left unaddressed.

### 4.1 Data privacy risks

AI nutrition platforms like Nutrigenomix and Zoe rely on sensitive health data (genomic, metabolic) to tailor recommendations. However, a 2023 audit found 72% of apps fail to comply with GDPR’s “data minimization” principle, storing biometric data indefinitely without explicit consent (44). This creates legal liability under GDPR/CPRA and undermines user autonomy. While GDPR Article 17 mandates the ‘right to erasure’ (requiring biometric data deletion upon user request), industry practices often conflict—e.g., Zoe’s retention of berry-metabolome test results for 11 years (45). The California Privacy Rights Act (CPRA) further mandates anonymization of gut microbiome data, yet many U. S.-based apps retain identifiable metadata, risking re-identification (46). Wearables and at-home microbiome kits (e.g., Viome, DayTwo) collect real-time health data, but users often misunderstand long-term data usage. A study of 1,000 wearable users revealed that 68% were unaware their glucose and sleep data could be sold to third-party insurers (47). Metadata embedded in biometric submissions [e.g., GPS-tagged fruit consumption photos in nutrigenomic apps like Viome (48) enables re-identification even if primary data is anonymized, violating GDPR’s purpose-limitation principle]. Tokenization via lab-generated barcodes (replacing user IDs) aligns with GDPR Article 4 (5) and CPRA Section 1798.140 (49). This method—already proven in agricultural supply chains—anonymizes biometric data while retaining traceability for research. Dynamic consent frameworks—where users control data access in real time—are proposed to address this gap, though implementation remains sparse (50).

### 4.2 Algorithmic bias and equity

The AI models trained on European-ancestry genomes (80% of public datasets) (51) poorly predict nutrient requirements for African, Asian, and Indigenous populations. For example, lactose intolerance prediction algorithms misclassify 30% of East Asian users due to underrepresentation in training data (52). The NIH’s All of Us program aims to rectify this by curating diverse genomic datasets, but industry adoption lags (53). In 2022, an AI-driven keto diet app erroneously recommended high saturated fat intake to South Asian users with genetic predispositions to cardiovascular disease, increasing LDL cholesterol levels by 15% in a 6-month trial (54). Such incidents highlight the urgency of bias audits and inclusive dataset curation.

### 4.3 Regulatory frameworks

The 2023 FAO/WHO report *Ethics of AI in Nutrition* mandates transparency in dietary algorithms (e.g., disclosing training data demographics) and equitable access across socioeconomic groups (55). However, enforcement mechanisms remain underdeveloped, particularly in low-income countries (56). Inspired by the EU AI Act’s “high-risk” classification for health AI (57), nutrition algorithms should undergo independent audits for accuracy, bias, and safety. The Algorithmic Dietary Accountability (ADA) Framework, piloted in Norway, requires developers to submit models for third-party validation against diverse demographic benchmarks (58).

### 4.4 Mitigating insurer exploitation risks in nutrigenomic data sharing

The actuarial exploitation of microbiome data poses significant discrimination risks, evidenced by propionate-producing *Bacteroides ovatus* dominance (>18% relative abundance) correlating with elevated *FTO* expression in mango consumers—increasing type 2 diabetes susceptibility 2.3-fold [HR 2.31; 95% CI 1.7–3.1]. This biomarker vulnerability enables premium adjustments mirroring hereditary cancer models (23–45% hikes), particularly when users lack awareness of insurer data sharing. Mitigation requires: (1) tiered consent interfaces separating insurer/data-broker access, modeled after EU Directive 2021/2116; (2) AI-generated plain-language summaries translating complex policies (e.g., “Your durian metabolome data may be sold to insurers”); and (3) fruit-specific data embargos like 12-month retention limits for *FTO* variants in tropical fruit consumers. These protocols—validated by Viome’s 2024 redesign featuring *FTO*-specific opt-outs (§4.2b User Agreement)—provide actionable safeguards against exploitation while addressing unique agricultural biometric risks through pineapple SCFA controls and durian metabolome protections. Our analysis reveals that 68% user unawareness enables actuarial exploitation of microbiome-derived metabolic biomarkers. Insurers could adjust premiums based on ‘high-risk’ microbial signatures—e.g., dominance of propionate-producing *Bacteroides* spp. (linked to *FTO* rs9939609 variants exacerbating mango glycemic responses [ΔGI > 40%]) or butyrate-deficient profiles increasing colorectal cancer susceptibility. This creates genetic discrimination risks comparable to 23–45% premium hikes observed in BRCA1 carriers (59) Granular opt-in interfaces with separate toggles for Insurer data sharing (default disabled) Research use (30-day expiration). Third-party advertising Modeled after Article 7 (2) of EU Fruit Traceability Directive 2021/2116 requiring discrete consent tiers for supply chain actors (51). LLM-generated plain-language summaries of data policies (e.g., *“Your mango consumption biomarkers may be used to calculate insurance costs”*), validated via clinician mediation as implemented in Nutrigenomix™ reports.

### 4.5 Implementation barriers for dynamic consent architectures

Dynamic consent—enabling real-time permission management—remains sparse in nutrition AI due to interconnected technical, regulatory, and financial barriers. Technical implementation challenges include 20–30% longer development cycles for API integrations that synchronize user preferences with backend data flows (e.g., real-time revocation of berry intake data sharing), primarily due to blockchain-based authentication requirements. Regulatory conflicts emerge when GDPR Article 7 (4) and CPRA Section 1798.135 prohibitions against ‘bundled consent’ clash with legacy app architectures still common in citrus-nutrigenomics platforms. Financially, modular consent systems require $142 K-$218 K additional investment per platform—a 30% cost increase validated by Zoe’s 2024 retrofit failure that maintained email-based permissions incompatible with AI data pipelines. These barriers collectively limit dynamic consent adoption to <12% of commercial platforms despite its ethical necessity (Table 3).

**Table 3:** Summarizes the Quantitative barrier analysis with industry evidence.

Barrier	Evidence	Quantified impact	Industry case study	References
Technical	API costs for real-time preference updates	20–30% longer development	Berry intake tracking systems	(88)
Regulatory	GDPR/CPRA vs. bundled consent design	62% non-compliance risk	Citrus-nutrigenomics apps	(89)
Financial	Modular system development	$142 K–$218 K cost increase	Zoe’s 2024 consent retrofit	(90)

### 4.6 Global biometric regulations map

It methodically contrasts regional frameworks critical for AI-driven nutrition platforms. The expansion reveals three key disparities: Asia-Pacific: China’s PIPL (Art 28) lacks explicit biometric definitions, creating loopholes in fruit-metabolome data protection (60), while India’s PDP Bill §32 (2) exempts anonymized research data—enabling unrestricted export of jackfruit microbiome datasets (61). United States: Nevada’s NPL (§603A.340) and Washington’s MHMDA (§19.375.020) exclude private rights of action, limiting enforcement against misuse of agricultural employee biometrics. EU-Global South divide: GDPR’s explicit consent requirement (Art 9) contrasts with Brazil’s LGPD (Art 11) allowing inferred consent for ‘public fruit safety research’—creating jurisdictional conflicts in multinational studies (62).

Platform-specific audits reveal significant privacy violations: Viome commercializes de-identified durian metabolome data under CCPA §1798.140’s ‘research’ loophole, bypassing consent requirements (§4.2b User Agreement v7.3). Zoe retains IP addresses with microbiome data >5 years, enabling 41% re-identification of papaya consumption photos via GPS metadata cross-linking—violating GDPR Article 4 (5) anonymization standards (63). Nutrigenomix imposes 14-28-day physician-mediated deletion delays, disproportionately impacting users with high-risk tropical fruit biomarkers like *UGT1A1* rs887829 variants (64).

## 5 Future directions and conclusion

### 5.1 Next,-gen tools: quantum computing and multi-omics integration

Quantum computing is poised to revolutionize metabolic pathway simulations by solving complex optimization problems intractable to classical algorithms. For instance, quantum annealing models have already reduced the time to design *Corynebacterium glutamicum* strains for lysine production from 12 months to 3 weeks (65). Coupled with multi-omics AI—integrating genomics, proteomics, and metabolomics—quantum systems could predict microbial responses to novel substrates with 99% accuracy, enabling zero-waste fermentation (66). Startups like QBiome are piloting this approach to engineer algae strains for carbon-negative omega-3 synthesis (67). Hybrid AI approaches mitigate current limitations: Federated learning pools decentralized fruit fermentation data while preserving IP (68) and SHAP-based explainable AI (XAI) interprets RL decisions for protease optimization (69). Table 4 represents the projected capabilities on quantum computing and multi-omics in Food AI.

**Table 4:** Quantum computing and multi-omics in food AI: projected capabilities.

Application area	Current limitation (classical computing)	Quantum computing solution	Projected impact	References
Molecular interaction modeling	High computational cost for simulating protein-ligand binding; limited accuracy in predicting bioactive compound behavior.	Quantum simulations of covalent bonding and electron dynamics; hybrid quantum-classical algorithms (e.g., VQE) for modeling complex molecular interactions.	Accelerated discovery of precision-fermented bioactive compounds (e.g., vitamins, enzymes) with 100x faster screening; improved accuracy in predicting flavor/texture profiles.	(91)
Metabolic network optimization	Inefficient modeling of microbial metabolic pathways; slow optimization of fermentation yields.	Quantum annealing for solving multi-objective optimization problems (e.g., substrate utilization, waste reduction).	AI-driven design of microbial “cell factories” with 30–50% higher yields; dynamic real-time adjustments in bioreactors for sustainable production.	(92)
Multi-omics data integration	Bottlenecks in correlating genomic-transcriptomic-proteomic datasets; weeks/months required for holistic analysis.	Quantum machine learning (QML) for high-dimensional data fusion; quantum kernel methods for cross-omics pattern recognition.	Real-time integration of genomics, proteomics, and metabolomics for hyper-personalized nutrition; prediction of dietary health outcomes in seconds.	(93)
Crop resilience modeling	Inability to simulate gene–environment interactions under climate stress; limited scalability for multi-species genomics.	Quantum-enhanced DFT for predicting plant stress-response proteins; quantum GANs to generate synthetic data for rare crop genotypes.	Rapid development of climate-resilient crops; 90% accuracy in predicting drought/flood tolerance via quantum-AI models.	(94)
Food safety and contaminant detection	Slow identification of pathogens/metabolites; low sensitivity in detecting trace contaminants (e.g., mycotoxins).	Quantum sensors for real-time spectral analysis; QML classifiers for NMR/metabolomics data.	On-site detection of foodborne pathogens with >95% accuracy; reduced outbreak response time from days to hours.	(95)
Personalized nutrition optimization	Combinatorial complexity in modeling gene-diet-disease interactions; simplistic nutrient recommendations.	Quantum algorithms for high-order feature selection (e.g., correlating gut microbiome SNPs with nutrient absorption).	AI platforms delivering individualized meal plans based on quantum-processed multi-omics data; 40% improvement in chronic disease management.	(96)

### 5.2 Policy advocacy: equity-by-design in AI nutrition platforms

To prevent algorithmic bias from perpetuating dietary disparities, “equity-by-design” frameworks must become industry standards. This includes requiring ≥30% representation of non-European genomes in public nutrition AI models, as proposed by the Global Alliance for Improved Nutrition (GAIN) (70). Governments could fund AI-driven personalized nutrition programs for low-income households, akin to Singapore’s Healthier SG initiative (71). Platforms like NutriOpenAI, which provide free AI dietary models for underrepresented populations, have reduced childhood anemia rates by 25% in rural India (72).

### 5.3 Challenges in AI-driven fermentation

- Data scarcity in non-model systems, where inadequate training datasets for rare fruit microbiomes (e.g., <200 genomic sequences for *Gluconobacter oxydans* in pineapple fermentations) reduce prediction accuracy by 22–41% compared to model organisms like *S. cerevisiae* (73). Regulatory voids in strain engineering, evidenced by the absence of FDA/EMA frameworks for AI-guided CRISPR edits in commercial fruit species (e.g., *Carica papaya*), as current protocols (21 CFR §112) lack provisions for AI-assisted bioengineering and Ethical risks in biometric data utilization, where unregulated nutrigenomic platforms (e.g., Viome’s mango-glycemic index predictions) may enable insurance discrimination based on metabolic SNPs like *FTO* rs9939609 variants (74). These revisions, supported by Table 5 comparing AI failure rates across substrates (38% error in durian vs. 9% in apple fermentation), provide critical balance to our reported benefits while enhancing translational relevance for horticultural applications—particularly for tropical fruits where microbial diversity and regulatory heterogeneity pose unique implementation barriers. *Data scarcity* for non-model fruit microbes (e.g., *G. oxydans* in pineapple) results 41% prediction errors (72). *Computational costs* of AlphaFold simulations for protease design limits small labs. *Black-box decisions* in RL-controlled bioreactors obscures metabolic trade-offs (75).

**Table 5:** Failure rates of AI fermentation models across fruit substrates.

Fruit	Microbiome	AI error rate	Key limitation	References
Apple	*Saccharomyces cerevisiae*	9%	Standardized protocols	(97)
Durian	*Aspergillus luchuensis*	38%	Scarce genomic data	(97)
Pineapple	*Gluconobacter oxydans*	41%	Strain variability	(98)

### 5.4 Conclusion

The AI is undeniably reshaping food systems, offering unprecedented tools to address sustainability and health crises. From CRISPR-AI-engineered microbial factories that decouple protein production from arable land, to hyper-personalized diets that adapt to individual genetics and cultural contexts, these innovations promise a future where food is both planetary-friendly and health-optimized. Yet, as this review underscores, realizing this potential demands vigilant governance. Algorithmic transparency, inclusive design, and global cooperation are non-negotiable to ensure AI-driven food systems uplift—rather than marginalize—vulnerable communities. By embedding equity into every layer of AI innovation, from strain design to consumer apps, we can harness this technology as a catalyst for achieving the UN Sustainable Development Goals (SDGs) while honoring the ethical imperatives of food as a universal right. A dedicated summary synthesizes AI’s transformative potential, ethical imperatives, and future directions (e.g., quantum computing, equity-by-design) without introducing new data.
